# Outpatient Management Protocol for Uncomplicated Diverticulitis: A 3-Year Monocentric Experience in a Tertiary Hospital

**DOI:** 10.3390/jcm13195920

**Published:** 2024-10-04

**Authors:** Marie Burgard, Alexis Litchinko, Jeremy Meyer, Christian Toso, Frédéric Ris, Vaihere Delaune

**Affiliations:** 1 Division of Digestive Surgery, Geneva University Hospitals, Rue Gabrielle-Perret-Gentil 4, 1211 Geneva, Switzerland; alexis.litchinko@hcuge.ch (A.L.); jeremy.meyer@hcuge.ch (J.M.); christian.toso@hcuge.ch (C.T.); frederic.ris@hcuge.ch (F.R.); vaihere.delaune@hcuge.ch (V.D.); 2 Department of Surgery, Cantonal Hospital of Fribourg Chemin des Pensionnats 2/6, 1752 Villars-sur-Glâne, Switzerland; 3 Transplantation and Hepatology Laboratory, Geneva Medical University, Rue Michel Servet 1, 1206 Geneva, Switzerland

**Keywords:** uncomplicated diverticulitis, outpatient management, treatment protocol

## Abstract

**Background/Objectives:** The management of acute uncomplicated diverticulitis (AUD) has shifted towards outpatient care in the last decade, challenging the traditional inpatient approach. We aimed to analyze the safety and feasibility of a structured outpatient treatment pathway for AUD in a tertiary hospital. **Methods:** We conducted a retrospective observational cohort analysis of patients who underwent outpatient management for AUD at the Geneva University Hospitals from 2019 to 2021. Patient demographics, selection criteria, treatment protocols, and outcomes were analyzed. **Results:** Two-hundred and twenty patients were included in the outpatient cohort. Four patients (1.8%) required hospitalization due to the failure of outpatient management, whereas the majority of patients (116 patients, 98.2%) experienced a successful resolution of their symptoms without hospitalization. In a univariate analysis, factors associated with treatment failure included elevated white blood cell counts at admission (14 G/l vs. 10.6 G/l, *p* = 0.049) and the first follow-up appointment, (10.7 G/l vs. 7.4 G/l, *p* = 0.011) and the presence of free air on their CT scan (25% vs. 2,3%, *p* = 0.033). In a multivariate analysis, the presence of free air was the only identified risk factor for unsuccessful outpatient management (*p* = 0.05). We observed high rates of follow-up compliance (99.1%). **Conclusion:** Under the condition of a warranted outpatient follow-up appointment and with adequate selection criteria, outpatient management appears to be an effective approach for most patients with AUD, emphasizing the importance of tailored therapeutic interventions and vigilant clinical assessments for optimal outcomes.

## 1. Introduction

Over the past decade, the paradigm of acute uncomplicated diverticulitis (AUD) management has undergone substantial reevaluation. This shift has been propelled by an expanding corpus of evidence suggesting that, for selected patients, outpatient management (predominantly characterized by the administration of oral antibiotics) can be as safe and effective as traditional inpatient care. Such outpatient strategies have demonstrated comparable outcomes in terms of complication rates, hospital readmission frequencies, and the necessity for surgical interventions, thereby challenging longstanding clinical dogma [1,2,3].

Contemporary clinical guidelines reflect this paradigm shift, advocating for the outpatient management of AUD in patients who do not exhibit sepsis, who are not immunocompromised, and who do not have contraindications such as an intolerance to oral intake or significant psychiatric or social impediments [4,5]. This patient-centric approach requires a nuanced understanding of individual patient profiles and the complexities of their conditions to ensure safe and effective treatment outside the hospital setting.

Parallel to the endorsement of outpatient management, there has been an emerging discourse surrounding the feasibility of non-antibiotic, observational, approaches for treating AUD. Recent studies have illustrated the safety and efficacy of such strategies, presenting them as viable alternatives that do not increase the risk of complications, necessitate hospital readmissions, or escalate to surgical interventions [6,7,8,9,10]. Despite the growing evidence base, the clinical community remains divided, with a substantial number of physicians expressing reservations about deviating from antibiotic treatments. This reluctance is often attributed to concerns over insufficient surveillance and follow-up mechanisms in outpatient settings, which could potentially compromise patient outcomes [11,12].

Moreover, the logistical challenges inherent in implementing non-antibiotic treatment pathways in outpatient settings underscore the need for comprehensive, evidence-based treatment algorithms. These protocols must account for rigorous patient selection criteria, ensure meticulous follow-up appointments, and foster patient education to mitigate the risks associated with treatment failures and complications [8,13,14,15].

Against this backdrop, our retrospective study seeks to meticulously analyze an outpatient management protocol for AUD, with a specific focus on treatment efficacy, failure rates, and patient compliance. By dissecting the outcomes associated with antibiotic management strategies within an outpatient context, this study endeavors to enrich the current understanding of AUD treatment modalities [8,16,17]. Furthermore, it aims to critically assess the operationalization of outpatient care for AUD, examining the interplay between patient selection, treatment efficacy, and follow-up compliance.

This investigation is based on the hypothesis that a well-structured outpatient management protocol for AUD can offer a safe, effective, and patient-centric alternative to inpatient care. It contributes to the ongoing discourse by evaluating the practicalities of implementing such a model in a real-world clinical setting, thereby offering insights that could guide future clinical practice and policy formulation [5].

The objective of this study was to evaluate our clinical protocol in terms of outpatient treatment failures, characterized by hospital admission, and their correlated risk factors. Adherence to the designated follow-up regimen among the patients and the occurrence rate of colorectal cancer within this demographic were analyzed as secondary outcomes.

## 2. Materials and Methods

This study is a retrospective, observational, cohort analysis conducted on a prospectively collected database at the Geneva University Hospitals, Switzerland, from May 2019 to June 2022. The selection of this period was based on the date of implementation of outpatient management of AUD in our institution and a safety analysis three years after implementation.

All patients receiving a diagnosis of acute uncomplicated diverticulitis, warranting an outpatient follow-up appointment, in the emergency department of the Geneva University Hospitals were prospectively included in the database. Uncomplicated diverticulitis was defined as the acute inflammatory state of a colonic segment, in the absence of any complications such as free fluid, moderate to major pneumoperitoneum, fistula formation, or abscesses, determined from the findings from computed tomography (CT) scans performed in the emergency department. For classification and staging purposes, we employed the Hinchey classification system [18]; Hinchey Ia was the classification used for AUD. These patients were eligible for outpatient management through our specific institutional protocol. Patients diagnosed with Hinchey Ia diverticulitis and a few amounts of extraluminal peri-colic air were classified as “Hinchey Ia+”, based on the knowledge that extraluminal air can constitute a risk factor for a disadvantaged short-term evolution [19]. Nevertheless, in the absence of other risk factors, those patients were also eligible for outpatient management.

**Figure 1:**
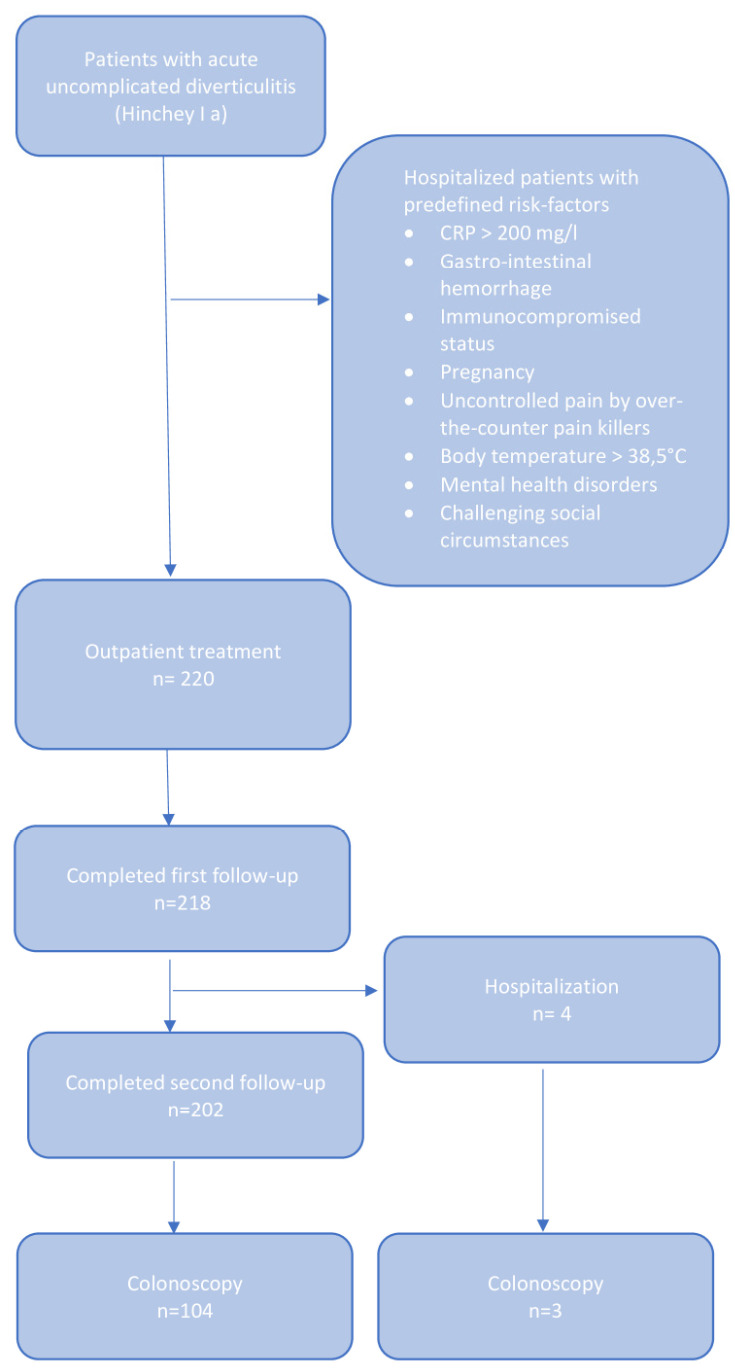
Inclusion flowchart.

Patients diagnosed with complicated diverticulitis, Hinchey Ib or higher, required direct hospitalization. All individuals exhibiting any of the following risk factors, based on existing studies [1,20], were also subjected to in-hospital treatment: lower gastrointestinal hemorrhage, a C-reactive protein (CRP) level exceeding 200 mg/L, mental health disorders or challenging social circumstances, an immunocompromised status, pregnancy, body temperature above 38.5 °C, pain not controlled by over-the-counter painkillers, a long weekend rendering close outpatient follow-up appointments impossible, or a recent episode of acute diverticulitis (within the last 3 months). The data for these patients were not used in this study. The outpatient treatment inclusion flowchart is illustrated in Figure 1.

**Figure 2:**
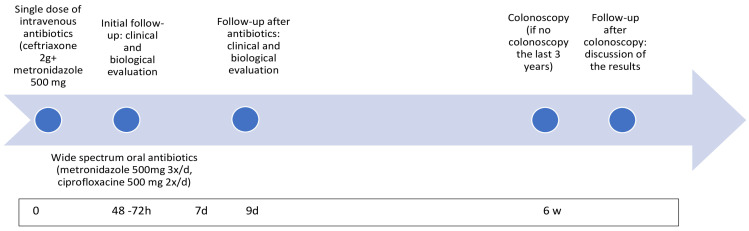
Outpatient treatment protocol.

The outpatient treatment protocol (Figure 2) started with a single dose of intravenous antibiotics administered in the emergency department, followed by a 7-day regimen of wide spectrum oral antibiotics. Due to local resistant bacterial strains, the intravenous regimen was usually comprised of Ceftriaxone/Metronidazole, and the oral regimen of Ciprofloxacin/Metronidazole. Additionally, patients received level I analgesics (paracetamol and non-steroid anti-inflammatory drugs if needed). An initial follow-up appointment for the clinical and laboratory evaluation was conducted at the Division of Digestive Surgery between 24 and 72 h after the initial consultation. A subsequent follow-up appointment for the clinical and laboratory assessment was scheduled 48 h after the antibiotic regimen’s conclusion. Furthermore, all patients lacking a recent colonoscopy (within the last 3 years) were advised to undergo this procedure at the 6-week mark, with a subsequent clinical consultation at the Division of Digestive Surgery to review the findings.

Should there be an adverse clinical or laboratory outcome during follow-up appointments, the protocol mandated a control CT scan and/or immediate hospital admission for further intravenous antibiotic administration. Patients unable to continue with oral medication were also admitted for inpatient care.

Medical and imaging data for the selected patients were retrospectively extracted. Key variables of interest included demographic data, clinical presentation including pain score, vital parameters, and biological markers at initial presentation, as well as biological markers at follow-ups and the result of the colonoscopy. Data confidentiality was maintained throughout the study, with all patient information being anonymized prior to analysis. Continuous variables were expressed as means with standard deviations; when variables did not follow a normal distribution, we used medians with the interquartile range. Continuous variables were analyzed using an independent *t*-test. Categorical variables were expressed as a number and percentage; they were analyzed using a Chi-squared test or Fisher’s exact test where appropriate. Univariate and multivariate Cox proportional hazard regression analyses were conducted to analyze the predictive factors of unsuccessful outpatient management. The results were expressed as hazard ratios with 95% confidence intervals. The statistical significance was defined as a two-tailed *p*-value ≤ 0.05.

A data analysis was performed using the Statistical Package for Social Sciences, version 29.0 (SPSS, IBM, Armonk, NY, USA.) We performed a complete-case analysis. No data were missing for patient demographics except for BMI (143/220 missing). At initial presentation, biological markers were complete and vital parameters were missing in 2/220 patients. At follow-up appointments, visit biological markers were missing in 5/220 patients.

All participants provided their written consent via an institutional consent form for further use of data for research purposes; this study received approval from the regional ethics board (CCER), BASEC-ID 2023-00661.

## 3. Results

Two hundred and twenty patients received outpatient management for acute diverticulitis. Among these patients, only four individuals (1.8%) required subsequent hospitalization during their follow-up period, while the vast majority (98.2%) experienced a successful resolution of symptoms under outpatient care. Causes leading to hospital admission or failure of outpatient management encompassed a lack of clinical improvement with oral antibiotic administration (*n* = 1), upper gastrointestinal bleeding (*n* = 1), abscess formation requiring radiological drainage (*n* = 1), and elevated CRP levels coupled with persistent abdominal pain (*n* = 1). Notably, none of the patients required emergency surgical intervention.

The demographic characteristics of the patient cohort are described in Table 1; both groups were comparable, albeit with a slight trend towards high blood pressure as a comorbidity in patients who were hospitalized during follow-up appointments (*p* = 0.07). A high proportion of patients in both groups had previously experienced at least one episode of acute diverticulitis (81% and 75%, respectively, *p* = 0.93).

The duration of symptoms and initial vital sign measurements were comparable across both patient cohorts, as shown in Table 2. However, patients experiencing unsuccessful outpatient treatment exhibited significantly elevated white blood cell (WBC) counts both at admission (14 G/l vs. 10.6 G/l, *p* = 0.05) and at the first follow-up appointment (10.7 G/l vs. 7.4 G/l, *p* = 0.05) compared to those with successful treatment outcomes. While C-reactive protein (CRP) levels at admission were similar between groups, individuals with unsuccessful outpatient management demonstrated a trend towards higher CRP values at the first follow-up appointment (100 mg/L vs. 54 mg/L, *p* = 0.07). Additionally, the presence of free air on their CT scan was markedly higher in patients experiencing unsuccessful outpatient treatment (25% vs. 2.3%, *p* = 0.006).

Univariate analyses found an elevated white blood cell count at admission (*p* = 0.049) and at the first follow-up visit (*p* = 0.011), as well as the presence of proximal free air (*p* = 0.033), which was associated with unsuccessful outpatient treatment. In the multivariate analysis, the presence of free proximal air was an independent factor associated with an unsuccessful outpatient treatment (*p* = 0.05), as shown in Table 3.

Follow-up appointments were adhered to by a significant majority of the patients. Two hundred and eighteen individuals (99.1%) attended the initial follow-up visit; completion of the second follow-up appointment was achieved by 202 patients (94%).

Of the total patient cohort, 107 individuals (49%) underwent a follow-up colonoscopy. Reasons for the non-completion of the colonoscopy were a recent normal examination within the past three years in 29% of patients, and the reason was undocumented in 71% of patients. A colonoscopic evaluation unveiled an underlying rectal neuroendocrine neoplasia in one patient (0.9%). Non-malignant polyps were found in 20/107 individuals (19%); there were no colorectal adenocarcinomas.

## 4. Discussion

The management of acute uncomplicated diverticulitis (AUD) has evolved significantly in recent years, reflecting a shift towards outpatient-based strategies. Our study adds to the growing body of evidence supporting the efficacy of outpatient management for AUD, when close monitoring is set in place. The overwhelming majority of patients in our cohort (98.2%) experienced a successful resolution of their symptoms without the need for hospitalization, and none needed urgent surgery, aligning with previous research highlighting the feasibility and safety of outpatient care [17]. This underscores the potential of outpatient pathways to alleviate healthcare burdens and costs associated with inpatient admissions, while maintaining favorable patient outcomes.

The success of outpatient management hinges on meticulous patient selection and adherence to structured treatment protocols. Our study adhered to stringent criteria for identifying candidates suitable for outpatient care, excluding individuals with complicated diverticulitis or significant risk factors necessitating hospitalization. Our selection of criteria justifying inpatient treatment was based on previously published papers [1,20]. Adherence to this protocol was associated with favorable outcomes, highlighting the importance of standardized approaches in optimizing patient care.

Despite the overall success of our outpatient management, a small subset of patients required subsequent hospitalization, necessitating an exploration of factors associated with treatment failure. Our analysis identified the presence of pericolic free air on their CT scan as a potential independent predictor of unsuccessful outpatient treatment. These findings corroborate previous research [21], suggesting a correlation between free air and disease severity in AUD, underscoring the utility of risk stratification and therapeutic decision making. Although several studies have shown a low failure rate of non-operative treatment for patients presenting with free pericolic air [22,23,24], to our knowledge, studies about the feasibility of outpatient treatment in this patient group are lacking. The updated WSES guidelines propose a non-operative strategy with antibiotic treatment for this specific group of patients, without, however, recommendations about inpatient or outpatient treatment [5]. In agreement with our findings, Costi et al. showed that many surgeons still promote inpatient treatment for patients with extraluminal free air [25].

Central to the success of outpatient management are robust follow-up and surveillance mechanisms to monitor treatment responses and detect complications promptly. Our study demonstrated high rates of follow-up compliance among the patients, with the vast majority attending scheduled appointments. This underscores the importance of patient involvement in their proposed health management method, and healthcare provider communication in facilitating the continuity of care and early intervention when necessary. The inclusion of routine colonoscopy in our follow-up protocol enabled the detection of one underlying neoplastic lesion (0.9%). This small percentage of underlying neoplastic lesions in uncomplicated diverticulitis is consistent with several studies [4,16,26,27]. A tailored approach regarding an endoscopy to avoid complications and limit costs therefore seems reasonable [8,28,29,30].

While our findings support the feasibility of outpatient management for AUD, several challenges and areas for future investigation warrant consideration. The logistic complexities of implementing outpatient protocols, including patient education, resource allocation, and interdisciplinary coordination, pose practical barriers to widespread adoption. In their recent snapshot study, Dalby et al. showed a higher adherence to current guidelines concerning outpatient management for AUD in centers that had previously participated in the AVOD trial, rather than in non-participating centers [16,31]. These findings underline that there are still barriers to changing the “that’s how we’ve always done it” mentality in many hospitals. Moreover, the optimal duration and composition of antibiotic regimens, as well as the role of adjunct therapies such as probiotics or dietary modifications, remain the subjects of ongoing debate and warrant further exploration. Additionally, the long-term outcomes and recurrence rates following outpatient management merit continued scrutiny, particularly in the context of the increased incidence of acute diverticulitis in a younger population and patients’ quality of life [32].

Our study is not without limitations. As a retrospective analysis, it is inherently subject to selection bias and data incompleteness, although we reached a high rate of completeness of follow-up data. Furthermore, the generalizability of our findings may be limited by the specific patient population and institutional practices represented in our cohort. Finally, more than half our patients did not have evidence of a follow-up colonoscopy in our files, limiting the fortuitous discovery of colorectal cancer in this population. We, therefore, cannot generalize that patients with uncomplicated diverticulitis have a 0.9% risk of cancer; this number could be over- or under-estimated.

Future prospective studies incorporating larger, more diverse patient populations and longer follow-up periods are needed to validate our findings and elucidate additional factors influencing treatment outcomes.

The high patient compliance in our study encourages us to pursue a non-antibiotic outpatient treatment regimen and analyze the long-term results.

## 5. Conclusions

Outpatient management appears to be an effective approach for the majority of patients presenting with acute uncomplicated diverticulitis, with a high success rate in this highly protocoled setting. However, careful monitoring is warranted, as a small proportion of patients may require subsequent hospitalization, particularly those with evidence of free air on their CT scan. These findings underscore the importance of vigilant clinical assessments to optimize outcomes in the management of acute diverticulitis in the outpatient setting. Further studies exploring risk stratification and optimal follow-up strategies are warranted to refine patient care and outcomes in this clinical context.

## Figures and Tables

**Table 1:** Demographic characteristics of the patient cohort.

	Outpatient *n* = 216	Hospitalization *n* = 4	*p*
Age, years, median (range)	57 (25–88)	56 (37–64)	0.404
Gender, *n* (%) F M	116 (53.7%) 100 (46.3%)	1 (25%) 3 (75%)	0.254
BMI, kg/m^2^, mean, (SD)	28.7 (±5.1)	26.5 (±7.1)	0.565
Comorbidities, *n* (%) HTA (%) Diabetes	70 (32.4%) 14 (6.5%)	3 (75%) 0	0.073 0.599
Tobacco use, *n* (%)	44 (20.4%)	1 (25%)	0.909
History of surgery for diverticulitis, *n* (%)	10 (4.6%)	0	0.660
Previous episodes, *n* (%) 0 1 2 3 4 5 6 7 8 9	41 (19.2%) 112 (52.3%) 27 (12.6%) 16 (7.5%) 12 (5.6%) 1 (0.5%) 3 (1.4%) 0 1 (0.5%) 1 (0.5%)	1 (25%) 2 (25%) 1 (25%) 1 (25%) 0 0 0 0 0 0	0.931
Episodes in the last 2 years, *n* (%) 0 1 2 3 4	176 (81.5%) 27 (12.5%) 9 (4.2%) 3 (1.4%) 1 (0.5%)	4 (100%) 0 0 0 0	0.925
Time since last episode, weeks mean (SD)	160.1 (±167.8)	198 (±59.4)	0.753

*n* = number, F = female, M = male, BMI = body mass index, kg = kilograms, m = meters, SD = standard deviation, and HTA = arterial hypertension.

**Table 2:** Initial vital signs, blood sample, and imaging parameters.

	Outpatient *n* = 216	Hospitalization *n* = 4	*p*
Time since beginning of symptoms, days, mean (SD)	2.7 (±2.1)	2.25 (±1.5)	0.668
HR, BPM, mean (SD)	89.7 (±14.8)	80 (±14.1)	0.196
Mean BP, mmHG mean (SD)	103.8 (±13.9)	96.3 (±14.5)	0.284
Temperature, °C, mean (SD)	36.9 (±0.7)	37.4 (±0.7)	0.288
Pain score, mean (SD)	4.3 (±2.5)	5.8 (±2.1)	0.225
Lc, mean (SD) - At admission - At first follow-up appointment	10.6 (±3.4) 7.4 (±2.3)	14 (±1.6) 10.7 (±2.1)	0.05 0.05
CRP, mean (SD) - At admission - At first follow-up appointment	66.5 (±43.9) 54.2 (±48.9)	73.5 (±60.1) 99.5 (±93.9)	0.755 0.073
Affected colon segment, *n* (%) - Right colon - Transverse - Left colon - Sigmoid	18 (8.3%) 1 (0.5%) 34 (15.7%) 163 (75.5%)	0 0 0 4 (100%)	0.731
Free air, *n* (%) - Proximal - Distant	5 (2.3%) 5 (100) 0	1 (25%) 1 (100%) 0	0.006

*n* = number, SD = standard deviation, HR = heart rate, BMP = beats per minute, BP = blood pressure, mmHG = millimeter mercury, C = Celsius, Lc = leucocyte, and CRP = C-reactive protein.

**Table 3:** Univariate and multivariate Cox regression of factors associated with unsuccessful outpatient treatment.

	Univariate	*p*	Multivariate	*p*
Time since beginning of symptoms, days	0.87 (0.47–1.61)	0.667		
HR, BPM	0.94 (0.87–1.03)	0.203		
Mean BP, mmHG	0.96 (0.89–1.04)	0.289		
Temperature, °C	1.17 (0.23–6.08)	0.850	1.07 (0.38–3.00)	0.902
Pain score	1.32 (0.83–2.11)	0.238		
Lc - At admission - At first follow-up appointment	1.42 (0.98–1.79) 1.71 (1.13–2.59)	0.049 0.011	1.16 (0.8–1.69) 1.60 (0.97–2.55)	0.436 0.092
CRP - At admission - At first follow-up appointment	1.01 (0.98–1.03) 1.01(0.99–1.03)	0.754 0.093	1.01 (0.975–1.03) 1.02 (0.98–1.01)	0.939 0.898
Affected colon segment - Right colon - Transverse - Left colon - Sigmoid	0.00 0.00 0.00 Ref	0.99 1.00 0.98		
Free air - No free air - Proximal free air	Ref 14.1 (1.24–159.89)	0.033	11.72 (0.67–206.28)	0.048

HR = heart rate, BPM = beats per minute, BP = blood pressure, mmHg = millimeter mercury, C = Celsius, Lc = leucocyte, CRP = C-reactive protein, and Ref = reference.

## Data Availability

Data generated or analyzed during this study are available from the corresponding author upon reasonable request.
